# Homœopathy

**Published:** 1882-08

**Authors:** A. B. Lyman

**Affiliations:** Baltimore


*FOR CONTENTS OF THIS NUMBER SEE PAGE 528.*

*THE*

### Independent Practitioner.

VOL. III.——*August, 1882.*——NO. 8.

### ORIGINAL COMMUNICATIONS.

*We are not responsible for the opinions expressed by contributors.*

ARTICLE I.

### HOMŒOPATHY.

BY A. B. LYMAN, M.D., BALTIMORE.

"What's in a name. A rose by any other name would smell as sweet."

Is is commonly supposed that there is nothing in a name.

Shakespeare does not say there is, or there is not. It is indeed doubtful whether he intended to convey the idea, by indirect implication, that a name amounts to nothing. He asks a question, but he does not answer it either in the affirmative or in the negative.

It is just as probable that he meant to express the opinion that there is a great deal in a name, as to commit himself to the view that there is nothing in a name.

So far as the expression *Homœopathy* is concerned, there can be no doubt but that the name of that arch-humbug and swindle has in it a powerful factor in its favor—that is, so far as it concerns the English-speaking people; the English folk, as Dr. Freeman would say. Why the English folk, above all others? Are they more easily cheated; have they less common sense, or less intelligence than the nations who speak the tongues of Cæsar or Arminius? That is the question. How is it to be answered?

The story is told, somewhere or other, of an old lady who was particularly impressed with the discourse of a certain reverend gentleman, because he made such frequent use in his sermon of, as she expressed it, "That blessed name, *Mesopotamia*," which she considered to be "so very comforting." Now had the clergyman made frequent repetitions of *Entre Rios*, or *Coblenz*, or *Interlaken*, it is very questionable whether the elderly gentlewoman would have derived so much comfort from their soothing sounds, although they have in different languages (or corruptions of them) significations by no means dissimilar to that of Mesopotamia. It is related of this same respectable female individual, that she never would, under any circumstances, take that abominable and disgusting medicine, *Tartar Emetic*; but that whenever she felt that she required anything of a character to cause easy vomiting she invariably made use of Antimonial Wine! What's in a name? Antimonial Wine, by any other name, will nauseate as well. But would the aged dame be willing to swallow it by any other name? As the oft-quoted bard has it, "That's the question." Had she known anything of philology, so far as the Grecian tongue is concerned, "That blessed name Mesopotamia" would have conveyed but litle comfort to her so far as its meaning is concerned, nor would she have scrupled to put herself outside of a dose of Tartar Emetic, in case her supply of Antimonial wine happened to run short, had her love of tongues enabled her to know what she was talking about, or her acquaintance with therapeutics placed her in a position to express any valid opinion concerning the merits of this or that remedial agent.

What proportion of the general public is any better prepared to speak with authority on subjects involving a knowledge of languages, derivations, and the healing art, than this same old lady, matters which they are, as a rule, totally ignorant of? Is it worth the physician's while to attempt to argue upon such topics with the average upstart, who thinks he knows all about medicine because he has read Dr. Somebody's book, any more than it would be productive of any result for a scholar to bandy words relative to the origin of irregular second aorists in the Greek language with a scout, or a gyp, or a stiefel fuchs, because the latter had waited on or cleaned the boots of Donaldson, Jelf, Porson, Wordsworth or Herrmann, or had helped a printer's devil to clean the type with which Liddell & Scott's Lexicon had been printed? And yet this is virtually just what many of the profession are doing every day, wasting their time, breath, and temper in futile discussions with half-educated boobies and dunces, both male and female, who think that when their opponent attempts to enlighten them with reference to the meaning of words, or their derivation and etymology, he is merely endeavoring to mystify them by using big words, in order to prove that black is white.

But why has this name such a charm for Angle ears? Hahnemann, the originator of this so-called system of medicine, was a German. In looking through his Greek lexicon for a combination expressive of the key-note of his idea, he lit upon *homoiopatheia.* Like *heliograph*, *lithograph*, *chromograph*, *telegraph*, *telegram*, *cryptogram*, *phonograph*, or *telephone*, the term he selected was designed to express a definite idea, the signification of which is included in the word he coined. Yet, how many are the words whose original meaning has been totally changed, or even lost altogether.

A prevaricator was once he who walked *wide, with his legs apart.* Now if we see a patient walking into our office with his legs apart, and he goes on to detail certain symptoms of a friend of his, and asks for something good to cure that friend; or if he tells a tale about having sat down on a water-closet seat where he feels sure some one, suffering from a venereal disease, must have just preceded him, or that he splashed himself in using a public urinal, and so contracted what he believes the profanum vulgus calls clap, and professors of the healing art gonorrhœa, we cease to regard him in the light of a prevaricator because he *walks with his legs apart*, but because he *talks wide* of the truth. It is more than probable that but few persons, even among comparatively well-informed people know what the original meaning of prevarication is.

How many are there who know what homœopathy means?

It is a well-known fact that this system of therapeutics has altogether lost credit and currency in the land of its inventor.

The Roman, the Teuton, the Slav and the Celt view it in no other light than that of an egregious and exploded humbug. The Angle alone, in the land of his adoption, and in the others which he has since settled by colonization, still continues to look upon it with a kindly eye. Why is this thus, or what is the reason of this thusness?

Let us for a moment reach down Worcester's Dictionary of the English Language and turn to the word *Home.* Ah! here it is. What does it mean? Is it hard to pronounce? Does its sound or association suggest anything of an unpleasant or repulsive character? Is it unfamiliar, or difficult to remember?

As spelled by Worcester, Homeopathic follows immediately after Home-made, and to the average reader recalls the idea of home.

Instead of being pronounced correctly in *five* syllables, ho-me-o-path-ic, it is commonly called home-o-path-ic, in four, and, etymologically, associated and connected, not with the Greek *homoios*, but with the English *home*. Here is the whole thing in a nutshell. Home! Sweet Home! The Home-o-path-ic physician provides you with medicine at Home, either at your own Home, or at his Home. A lady of my acquaintance, a confirmed home-o-pathist, I cannot say homœopathist (for that would not express her ideas concerning the matter), in conversation endeavored to convince me that castor oil is a strictly homœopathic remedy, and, in support of her position, strenuously contended that it must be home-o-pathic because a *domestic* remedy. Is it worth while arguing with such people, when the logical fallacy anticipitas medii is constantly cropping up? With us homœopathy means, if it means anything, a system of medicine founded on the dogma that similia similibus curantur, or like is cured by like. Vide Webster's definition of homœopathy. With our opponents in the argument, it means a method of practice where the remedies are obtained at the home of the physician, kept on hand for use at one's own home, or even made at home; "*home-made*," according to Worcester, signifying, "*made at home*," "*of domestic manufacture.*"

Not long ago a lady patient assured me that a certain physician was a homœopath, and in refutation of doubts expressed by me in the matter, assured me that he made all his own medicine at *home*.

What the public understand by a home-o-pathic physician, or that one might sincerely believe in the doctrine of similars, so far as relates to therapeutics, is easy of comprehension. The homœopathic manuals for domestic practice, however, speak of something much more wonderful, and that is a homœopathic surgeon.

We might be able to conceive of a man being a homœopathic physician, that he verily believed and practiced the tenets of Hahnemann, but when it comes to a homœopathic surgeon, that is a creature to ordinary mortals incomprehensible.

One of these manuals, just referred to, gives most minute and elaborate directions what to do in a number of injuries, more or less serious. The advice and information imparted are excellent until the sentence is reached, "*then send at once for the nearest homœopathic surgeon.*" What the h. s. does upon his arrival is best known to himself, but if he knows anything about surgery he most likely does very much the same as any of the "*allopathic*" surgeons would do under similar circumstances. It is difficult to bring oneself to imagine for one moment that he would procede * to treat the case on strictly homœopathic principles. No doubt he has shrewdness enough to make use of every domestic appliance within reach, and also to call particular attention to his doing so, with the view of impressing upon by-standers that he is a home-o-pathic surgeon.

Now if he were a consistent homœopathic surgeon he would, as a matter of course, apply such treatment as would produce results *similar* to the condition he was called upon to cure. It would be superfluous to give illustrations of what he might and ought to do in order to be "homœopathic." For instance, in the case of a fracture of the femur about the middle third, would he apply such dressings and apparatus as would produce a similar fracture in a sound limb? The very suggestion conveys its own answer. He would not dare to treat the case, or a wound, gun-shot or other, on homœopathic principles, for fear of criminal prosecution for having maimed his patient, perhaps for life. Just imagine a surgical case treated homœopathically!

Another anomaly is the homœopathic practitioner of midwifery.

It is hardly worth while to attempt to explain such an evident paradox as homœopathic obstetrics. Enough has been said to plainly demonstrate the fact that, etymologically and derivatively, there is nothing whatever in the name, so far as actual practice goes. While there are plenty of home-o-pathic doctors in the world, particularly where the English language is spoken, those who practice homœopathy are extremely scarce. The name is retained because it pays to stick to it, and because it is doubtful whether any other could be devised which would sound as sweet to Angle ears, although carrying no weight with Romans, Teutons, Slavs, or Celts.

To show how utterly inconsistent names sometimes are with the business they are supposed to represent, I may by way of illustration mention that in the city of Denver, in Hollady Street, between 17th and 18th Streets, is a pretentious establishment bearing on its front, in heavy but well-executed black letters at least eighteen inches in height, the following sign: "Orthopædic Institute for Horses."

* Etymological spelling; usually spelt *proceed.*

There are about as many people who know what *orthopædic* means as there are who know what *homœopathic* means. Both terms present to their minds the idea of some special, new and improved method of treatment.

The only cases I ever heard of where homœopathy was actually made use of were in the practice of an abortionist. It was his habit to subject nearly every one of his lady patients to a preliminary course of homœopathy, having first secured his fee in advance. I never heard of a case in which his homœopathic method was successful in producing miscarriage, while, on the contrary, I did hear of one in which a young woman, who had consulted him with reference to painful menstruation, actually became pregnant after undergoing his strictly homœopathic mode of treatment for a short period.

